# Optical metasurfaces for generating and manipulating optical vortex beams

**DOI:** 10.1515/nanoph-2021-0746

**Published:** 2022-01-10

**Authors:** Hammad Ahmed, Hongyoon Kim, Yuebian Zhang, Yuttana Intaravanne, Jaehyuck Jang, Junsuk Rho, Shuqi Chen, Xianzhong Chen

**Affiliations:** School of Engineering and Physical Sciences, Heriot-Watt University, Edinburgh EH144AS, UK; Department of Mechanical Engineering, Pohang University of Science and Technology (POSTECH), Pohang 37673, Republic of Korea; School of Physics and TEDA Applied Physics Institute, Nankai University, 94 Weijin Road, Tianjin, 300071, China; Department of Chemical Engineering, Pohang University of Science and Technology (POSTECH), Pohang 37673, Republic of Korea; POSCO-POSTECH-RIST Convergence Research Center for Flat Optics and Metaphotonics, Pohang 37673, Republic of Korea

**Keywords:** nonlinear metasurfaces, OAM holography, OAM multiplexing, OAM sorting, OAM superposition, optical metasurfaces

## Abstract

Optical vortices (OVs) carrying orbital angular momentum (OAM) have attracted considerable interest in the field of optics and photonics owing to their peculiar optical features and extra degree of freedom for carrying information. Although there have been significant efforts to realize OVs using conventional optics, it is limited by large volume, high cost, and lack of design flexibility. Optical metasurfaces have recently attracted tremendous interest due to their unprecedented capability in the manipulation of the amplitude, phase, polarization, and frequency of light at a subwavelength scale. Optical metasurfaces have revolutionized design concepts in photonics, providing a new platform to develop ultrathin optical devices for the realization of OVs at subwavelength resolution. In this article, we will review the recent progress in optical metasurface-based OVs. We provide a comprehensive discussion on the optical manipulation of OVs, including OAM superposition, OAM sorting, OAM multiplexing, OAM holography, and nonlinear metasurfaces for OAM generation and manipulation. The rapid development of metasurface for OVs generation and manipulation will play an important role in many relevant research fields. We expect that metasurface will fuel the continuous progress of wearable and portable consumer electronics and optics where low-cost and miniaturized OAM related systems are in high demand.

## 1 Introduction

Inspired by hydrodynamic vortices, the concept of optical vortices (OVs) was created by Coullet et al. in 1989 [1]. An OV beam possesses a phase singularity with a spiral wavefront and carries quantized orbital angular momentum (OAM). OVs can be generated by spiral phase plates, which can directly impose a vortex structure on a plane wave by linearly varying the optical path length around the circumference of an optical device. Benefiting from peculiar optical features and the extra degree of freedom for light manipulation, OVs have been widely investigated as promising resources and have been applied in many research fields, including quantum science [2], particle trapping [3], optical communications [4, 5], and high-resolution lithography [6], [7], [8]. Recently, vector OVs have stimulated considerate interest since they both have inhomogeneous polarization distribution in the transverse plane and carry OAM. For example, a radially polarized beam can be more sharply focused and give rise to a centered longitudinal field, which has been applied in the higher resolution lithography. Spatial light modulators and liquid-crystal q-plates [9, 10] have been proposed to generate OV beams. However, the limitations of poor resolution, large volume, high cost, and lack of design flexibility still need to be overcome for practical applications. There are many challenges in building devices that are compact, efficient, and integrable.

Driven by device miniaturization and system integration, there is huge interest in developing ultrathin (light wavelength scale) and lightweight planar optical devices with novel functionalities that cannot be obtained with conventional optical elements. Optical metasurfaces are planar nanostructured interfaces and have recently attracted tremendous interests due to their unprecedented capability in the manipulation of the amplitude, phase, and polarization of light at subwavelength scale [11], [12], [13], [14], [15]. The emergent optical metasurface-based flat optics has revolutionized design concepts in photonics, providing a new platform to develop unusual ultrathin optical devices, including metalenses [16], [17], [18], [19], [20], holograms [21], [22], [23], [24], colour filters [25, 26], and so on. In addition, nonlinear optical metasurfaces have been applied to second [27], third [28], and high harmonic generation [29], and optical frequency mixers [30, 31]. Nonlinear metasurfaces for holography [32, 33], spin-orbital interactions of light [34], metalenses [35], and image encryption [36] have been experimentally demonstrated. Optical metasurfaces have shown much promise for the generation and manipulation of structured beams. Generating and manipulating these structured beams with ultrathin optical devices has been a hot topic in recent years since the advent of optical metasurfaces, which have opened a new avenue to develop unusual ultrathin optical devices for the realization of OVs at the nanoscale.

We concentrate on the applications of metasurfaces in generating different types of multifunctional OVs and manipulating these beams. With the promising capabilities and potential for low-cost and miniaturized OAM related systems, we hope that metasurfaces will fuel the continuous progress of wearable and portable consumer electronics and optics. The review is organized as follows. In Section 2, we focus on the fundamentals of OVs, existing challenges, and the advantages of optical metasurfaces. In Section 3, we emphasize the methodologies utilized to generate different types of multifunctional OVs based on metasurfaces. In Section 4, we review the current progress on the optical manipulation of OVs, including OV superposition, OV sorting, OV multiplexing, OV holography, and nonlinear optical metasurfaces for OV manipulation. In Section 5, we give concluding remarks and an outlook on future research directions.

## 2 Fundamentals

OVs carrying OAM naturally exhibit twisted/helical wavefronts. The degree of twist/helicity is determined by the topological charge (*l*) of the OV beam [37]. There are number of solutions to the Helmholtz wave equation, which can result in kinds of beams that carry OAM. Examples of these solutions with an OAM include hypergeometric-Gaussian modes [38], Laguerre–Gaussian (LG) modes [39], Bessel modes [40], https://www.spiedigitallibrary.org/journals/optical-engineering/volume-59/issue-4/041205/Generation-and-decomposition-of-scalar-and-vector-modes-carrying-orbital/10.1117/1.OE.59.4.041205.full - r8 Ince–Gaussian modes [41], and Mathieu modes [42]. LG modes are a set of solutions to the paraxial wave equation in cylindrical coordinates (*r*, *θ*, *z*). They are mainly characterized by two parameters, *l* and *p* which are topological charge, number of radial nodes, respectively. The corresponding expression in the cylindrical coordinates is given as:

(1) $${\text{LG}}_{p,\;l}\left(r,\theta ,\;z\right)=\sqrt{\frac{2p!}{\pi \left(p+\left|l\right|\right)!}}\frac{1}{w\left(z\right)}{\left[\frac{r\sqrt{2}}{w\left(z\right)}\right]}^{\left|l\right|}\exp\left[-\frac{r^{2}}{w^{2}\left(z\right)}\right]L_{p}^{\left|l\right|}\left[\frac{2r^{2}}{w^{2}\left(z\right)}\right]\exp\left(il\theta \right)\times \exp\left[\frac{ikr^{2}z}{2\left(z^{2}+z_{R}^{2}\right)}\right]\exp\left[-i\left(2p+\left|l\right|+1\right)\tan^{-1}\left(\frac{z}{z_{R}}\right)\right]$$

where

(2) $$w\left(z\right)=w_{0}\sqrt{1+{\left(\frac{z}{z_{R}}\right)}^{2}},\;z_{R}=\frac{\pi w_{0}^{2}}{\lambda }$$

**Figure 1:**
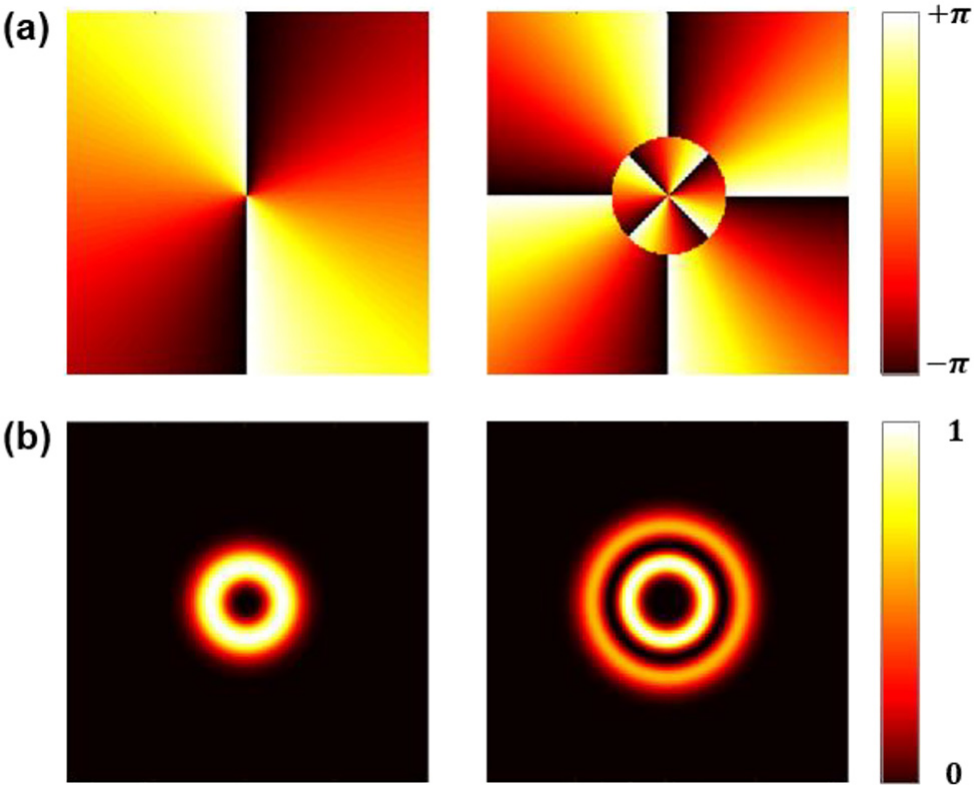
Typical LG beams depiction. (a) Phase profiles for l=2, p=0 (left)and l=4, p=1(right). (b) Corresponding intensity profiles.

In the above equations, *w*_*z*_ denotes the beam radius at *z*, while *w*_0_ is the beam radius at *z* = 0 (known as waist radius). The parameter *z*_*R*_ is the Rayleigh range of Gaussian envelope, and $\left(2p+\left|l\right|+1\right){\text{tan}}^{-1}\left(z/z_{R}\right)$ corresponds to the Gouy phase. Different LG beams can be determined by altering *l* and *p*. All LG beams represent a set of orthogonal basis vectors that can correspond to any spatial beams. The intensity and phase distributions with different *l* and *p* values are depicted in Figure 1. The radius of the doughnut-shaped beam will increase when *l* increases, while the *p* will lead to a change in the number of rings.

## 3 OV generation

**Figure 2:**
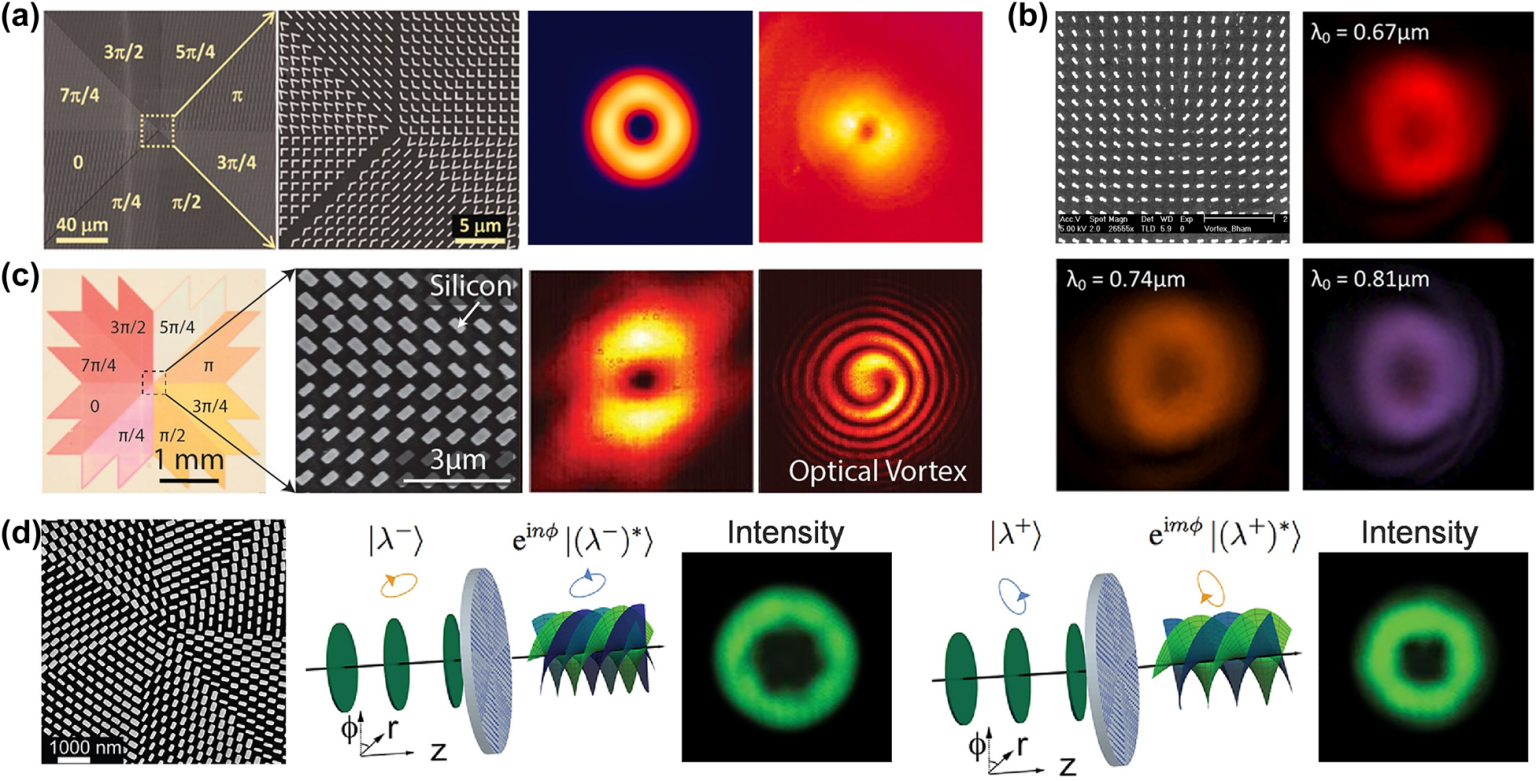
Metasurface for OV generation. (a) Left: Scanning electron microscopy image (SEM) image of a fabricated plasmonic metasurface that generates an optical vortex. The unit cells are arranged in such a way that phase shift varies azimuthally from 0 to 2*π*. Right: Simulated and measured far-field intensity pattern of an OV with *l* = 1 [11]. (b) Top left: SEM image of the fabricated gold nanopillar based OV generator. Measured intensity distribution of the vortex beam patterns at 670 nm (top right), 740 nm (bottom left), 810 nm (bottom right) [44]. (c) Left: SEM image of dielectric metasurface based spiral phase plate. Right: Measured far-field intensity and interference pattern of a OV (*l* = 1) [51]. (d) Metasurface based J-plate for arbitrary spin-orbital angular momentum conversion [52].

Capasso and colleagues [11], for the first time, demonstrated the plasmonic metasurface consisting of V-shaped nano-antennas for the generation of OVs (Figure 2a). This type of design leads to a novel ultrathin phase plate with significantly high resolution. Such metasurface introduced a helical phase shift at the interface with respect to incident linearly polarized (LP) plane wave, generating an OV with *l* = 1. The OV has donut-shaped intensity ring in the cross-section. A dark region can be seen at the centre, which represents a phase singularity [43].

Subsequently, a plasmonic metasurface for generating an abrupt phase change and controlling the wavefront for circularly polarized (CP) light at the visible and near-infrared wavelengths has been proposed [44, 45]. The metasurface consists of an array of metallic nanorods with the same geometry but spatially varying orientations (Figure 2b). When a beam of CP light is incident onto a dipole antenna, the scattered wave is partially converted into the opposite handedness of CP light with a phase change determined solely by the orientation of the nanorod. At relatively small incident and observation angles, the scattered field can be approximated as:

(3) $$E_{rad}\approx c\alpha_{e}\;k^{2}e^{ikr}\left[\frac{\text{cos}\theta \text{cos}\zeta +1}{4}E_{u}^{\sigma }+\frac{\left(\text{cos}\theta +1\right)\left(\text{cos}\zeta +1\right)}{8}E_{u}^{-\sigma }e^{+\sigma i2\phi }\right]$$

where *r* is the distance from the antenna, *k* is the wave vector, and *α*_*e*_ is the dipole moment of the antenna. *θ* and *ζ* are, respectively, the incident and observation angles, and $E_{u}^{\pm \sigma }=\left(\text{cos}\zeta e_{x}\pm \sigma ie_{y}-\sin\;\zeta e_{z}\right)$. Equation (3) shows that, within a certain range of incident angle around the surface normal, a CP beam is primarily scattered into waves of the same polarization as that of the incident beam without phase change, and waves of the opposite CP with a phase change *ψ* = 2*σϕ* twice the angle formed between the dipole and the *x*-axis, where *σ* = ±1 correspond to the helicity of right-handed circularly polarized (RCP) and left-handed circularly polarized (LCP) incident light. This phase change can be understood in the context of the Pancharatnam−Berry (PB) phase that is acquired when the polarization state of light is changed [46, 47]. Figure 2b shows the plasmonic interface that is created by arranging the dipoles with spatially varying orientation angles that rotate 180° when the location of the dipoles rotates by 360° around the singular point. The interface introduces a spiral-like phase shift with respect to the planar wavefront of the incident light, creating a vortex beam with a topological charge *l* = 1. Because the azimuthal phase profile is abruptly introduced through the interaction with the dipole plane, the screw-like phase profile characteristic of the vortex beam can be created over a negligible propagation distance. The measured vortex beams at different incident wavelengths are shown in Figure 2b.

Later on, to overcome the problem of low coupling efficiency and Ohmic losses caused by plasmonic metasurface, a broadband dielectric metasurface [48], [49], [50] has been proposed by utilizing silicon (Si) nano-resonators patterned above a silver layer [51]. The underlying physics behind the high efficiency is the excitation of Mie resonances inside the low aspect ratio nano-resonators. Each resonator acts as a half-waveplate with approximately unity reflectance and over 98% polarization conversion efficiency with a bandwidth of 200 nm. By varying the dimensions of resonators (e.g., length, width), the complete 2*π* phase coverage of the reflected cross-polarized light was acquired upon the illumination of LP light (Figure 2c).

Recently, a novel optical device has been proposed to demonstrate the conversion of arbitrary orthogonal polarizations to distinct OAM states. This shows a new degree of freedom in which one can precisely control polarization and phase using subwavelength nanostructures [52, 53]. Due to its ability to encode any arbitrary input spin angular momentum (SAM) to two arbitrary output total angular momentum (TAM) states, the authors called this device a J-plate. The working principle of a device is centered on the modulation of both the propagation phase and the PB phase. This gives an additional degree of freedom to the PB phase to be extended from CP states to any orthogonal polarization states. Each state, therefore, can hold an arbitrary OAM state. For instance, Figure 2d shows that two orthogonal elliptical polarization states of an incident wave can carry distinct OAM states with *l* = 3 and 4. Recent works on optical metasurfaces for OV generation are available in the references [54], [55], [56], [57], [58].

## 4 OV manipulation

The unprecedented capability of optical metasurfaces in light control provides a compact platform not only to generate OVs but also to manipulate these beams in a desirable manner, including superpositions, sorting, multiplexing, holography, and nonlinear metasurfaces for OV manipulation. OV superposition can generate the interference pattern at far field, allowing for polarization detection. OV sorting can efficiently sort the input OV beam, depending on its topological charge and advancing the identification of OV modes. OAM multiplexing can generate different OV states in separate channels. OV holography adds an additional degree of freedom in the hologram using OV as an information carrier. Recently, OV manipulation has also been extended to nonlinear optical metasurfaces. Although the fundamentals in these subsections are closely related, they have different emphases.

### 4.1 OV superposition

**Figure 3:**
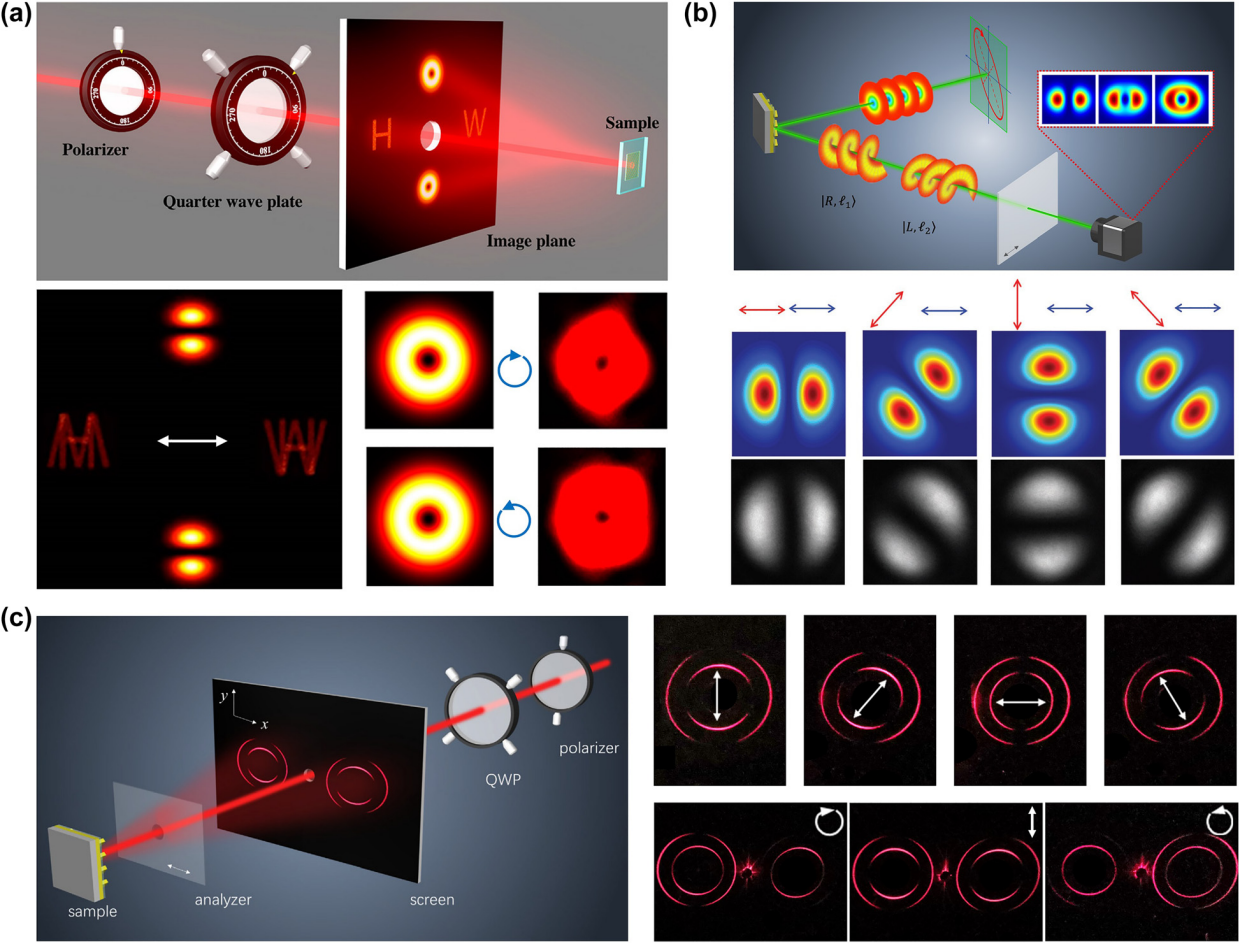
Metasurface for OV superposition. (a) Top: Schematic illustration of simultaneous control of hologram and superposition of OV [59]. Bottom: Simulation and experimentally measured results for LP, RCP, and LCP incident light at 640 nm [59]. (b) Top: Schematic of polarization detection system using light’s OAM [60]. Bottom: Measurement of major axis for the superposition of *l* = +1 (1st row) and *l* = −1 (2nd row) [60]. (c) Left: Schematic for generation and manipulation of ring OAM beams. Incident light with various polarization states is generated by a QWP and a polarizer [68]. Right: Simulated and measured intensity distributions for the superposition of OV beams upon the illumination of incident light with different polarizations.

To meet the growing requirement of system integration, it is of great importance and interest to develop ultrathin optical devices that can integrate multiple functionalities into one device while preserving their independent functionalities. An ultrathin optical device has been demonstrated to simultaneously realize polarization-controllable hologram and superposition of OAM in multiple channels (Figure 3a) [59]. By continuously controlling the polarization state of the incident light, the polarization-dependent holographic images in two channels along one direction and the manipulation of OAM superposition in two channels along the other orthogonal direction are realized. Not only the superpositions of OAM states but also the dynamic change of the holographic images can be manipulated at the same time. The simulated and measured intensity profiles of the OAM superposition are given in Figure 3a for various polarization states (LP, RCP and LCP) of the incident light. By continuously changing the polarization state of the incident light, the evolution process of the intensity distribution can be observed after the resultant OAM beam passes through the rotating polarizer (before the sample). When the LP light is incident onto the metasurface, the resultant output beams are the superposition of |R,l=1⟩and |L,l=−1⟩ with equal power.

Afterward, the technique of superposition was used in polarization detection [60]. Traditional polarization detection systems include many polarization elements, e.g., polarizers, waveplates, polarization modulators, leading to large volume and high cost [61]. Due to their ultrathin nature and high resolution, optical metasurfaces have become a strong candidate for polarization detection. Optical metasurfaces have been used in various polarization detection systems, including CP detection [23, 62], full polarization measurement [63], [64], [65], and polarimetric imaging [66, 67]. Figure 3b shows the schematic of the recent approach of the metasurface for polarization detection. When a light beam with unknown polarization shines on a reflective optical metasurface that encodes the following phase distribution ϕ(x,y):

(4) $$\phi \left(x,y\right)=\arg\left(E_{1}\;\exp\left(i\left(l_{1}\theta +\Delta \phi_{off}\right)\right)+E_{2}\;\exp\left(i\left(l_{2}\theta -\Delta \phi_{off}\right)\right)\right)$$

where *E*_1_ and *E*_2_ represent the amplitude components of two OAM beams with topological charges of *l*_1_ and *l*_2_, respectively. *θ* is the azimuthal angle and Δ*ϕ*_*off*_ is the phase difference between neighboring pixels to generate a phase gradient along the *x*-direction, which can introduce off-axis deflection for the OAM mode of interest. The emitted light generates the superposition of two OAM states, which passes through an analyzer (linear polarizer), whose transmission axis is fixed along the *x*-direction. The interference patterns collected by a CCD camera are closely related to the polarization state of the incident light. Based on the analysis of the intensity distribution, the polarization state is directly measured. The direction of the major axis and ellipticity of incident light can be measured by the intensity distribution of the superpositions of OAM states with the same topological charges and opposite signs. The handedness of incident light is measured by the distance of two maximum intensities of the superpositions of OAM states with different topological charges and opposite signs. Figure 3b (bottom) shows simulated and experimentally observed superpositions of OAM states with *l* = +1 and *l* = −1. The polarization direction of the incident linearly polarized light and the direction of the polarizer’s transmission axis in front of the CCD camera are, respectively, denoted by the red and blue double-headed arrows.

A riveting approach has been experimentally demonstrated to generate the ring OAM beams through a single metasurface [68]. By varying the polarization state of the incident light, the superposition between the generated ring OAM beams can be continuously tuned. A perfect vortex beam is the Fourier transform of a Bessel Gaussian beam, which can keep the size of the intensity pattern while the topological charge varies [69]. A single metasurface can generate perfect vortex beams by combining the functionalities of a vortex beam generator, axicon, beam deflector, and a phase profile of spherical wave for a Fourier transform lens [69, 70]. By contrast, the phase term for the Fourier transform lens is excluded in this metasurface. Therefore, due to the absence of a lens, the resulting ring OAM beam diameter change with the observation plane at different distances, allowing for the ring to be observed visually from the observation plane far enough away. Note that the radius of the ring only depends on the axicon period, thus the ring OAM beams in this work stay constant with different topological charges, enabling the superposition of the ring OAM beams in far-field observation [71, 72]. Figure 3c shows the schematic of the experimental setup based on an ultrathin metasurface to generate ring OAM states. A polarizer and a quarter-wave plate (QWP) are used to control the polarization state of the incident light. The superposition of ring OAM beams from the metasurface is displayed on a screen after passing through an analyzer.

### 4.2 OV sorting

OV detection has been mainly based on the unique features of OAM-distinct interference patterns. However, the interference pattern-based OAM detection is conducted in the far-field, which requires bulky optical setup. Moreover, detecting the topological charge of OVs by observing the interference pattern is inefficient for OAM identification, hindering the application of the OV systems in optical communications and signal processing. Recently, with the emergence of on-chip plasmonic nanostructure [73, 74] and dielectric metasurface [75], [76], [77], compact OAM detection method by sorting OAM modes has been proposed. The topological charge OVs is directly detected through OV sorting methods by measuring the position of the sorted beam.

**Figure 4:**
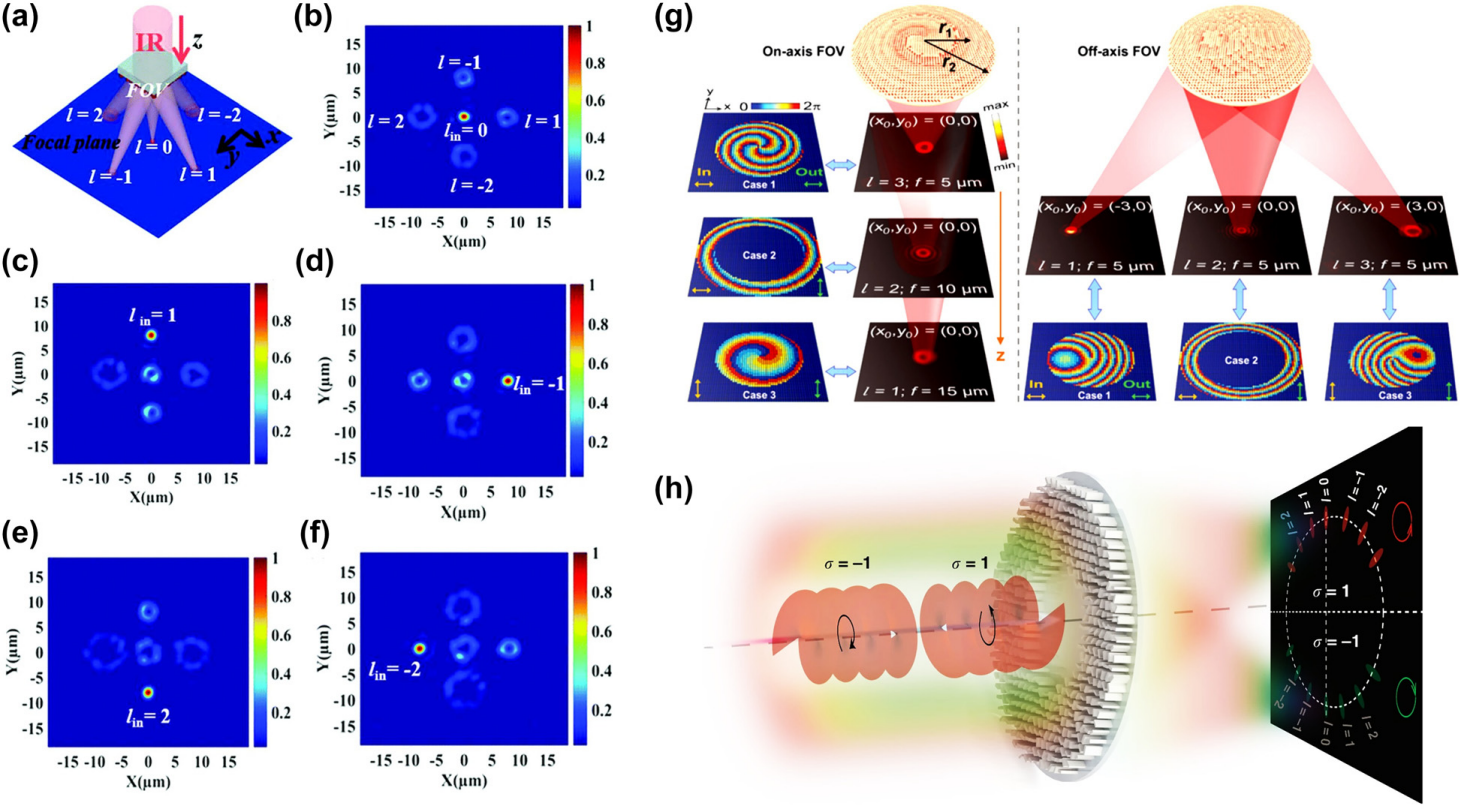
Metasurface for OV sorting. (a) Schematic of the multiple OV generator. The incident light is sorted into five OVs with different *l*. (b–f) The corresponding intensity distributions. The incident light is impinged with (b) *l* = 0, (c) *l* = 1, (d) *l* = −1, (e) *l* = 2, (f) *l* = −2 [75]. (g) Left: On-axis OVs with *l* = 3, 2, and 1 at *f* = 5, 10, and 15 μm. First column shows the calculated phase distributions. The 2nd column is far-field intensity patterns. Right: Off-axis OVs with *l* = 1, 2, and 3 at *f* = 5 μm. 1st row shows the far-field intensity patterns. The 2nd row is the corresponding calculated phase distributions [76]. (h) Schematic of dielectric metasurface that combines the PB phase and propagation phase for simultaneous OV and SAM sorting. OVs with various incident spins are translated into focusing spots on the screen with two separated halves [77].

Recently, the interaction between the incident beam and the focused optical vortex has been performed [75]. By looking at the specific position of the focal spot, the topological charge of OV can be identified. The incident beam with topological charge *l*_*in*_ interacts with the metasurface that has *l*_*m*_. The output beam will carry the OAM as *l*_*out*_ = (*l*_*in*_ + *l*_*m*_). Here, a predesigned metasurface with known OV generation function is impinged by incident light with topological charge varying from −2 to 2. The corresponding intensity distributions 30 μm away from the metasurface are shown in Figure 4a–f. When the *l*_*out*_ is zero, it implies the annihilation of OV because the metasurface compensates the ‘*l*’ with the opposite sign ‘−*l*’. Thus, the resultant solid spot (annihilated OAM) at the focal plane is used as a reference to indicate the topological charge of an incident OV.

In the due course, a novel metasurface with twofold polarization and trifunctionality has been proposed [76]. The metasurface is composed of two different meta-atoms, i.e., polarization maintaining meta-atom, and polarization converting meta-atom. The polarization maintaining meta-atom independently manipulates the phase shifts for orthogonal input polarizations, while the polarization converting meta-atom simultaneously performs polarization conversion and phase manipulation. By interleaving these two meta-atoms, three independent functions can be encoded to the metasurface [78]. The on-axis and off-axis sorting of focused OV can be achieved where OV is focused at three different on-axis and off-axis focal points with OAM beam with three different topological charges, as shown in Figure 4g. In on-axis design, metasurface transform an impinging plane wave into three distinct focused OVs (with *l* = 3, 2, 1) at focal planes *z* = 5, 10, and 15 μm, respectively, by placing polarization converting meta-atom in a circular fashion with radius *r*_1_ and placing the polarization maintaining meta-atom in a circular ring with a radius extending from *r*_1_ to *r*_2_. The corresponding phase profiles are illustrated on the left side (1st column) of Figure 4g. The left side (2nd column) of Figure 4g depicts the intensity pattern in *xy*-plane at the focal planes under specified incident polarization states. On the other hand, the off-axis design renders three horizontally separated focused OVs at the same focal planes (z = 5 μm). The phase profiles and the corresponding donut-shaped intensity distributions are illustrated in Figure 4h (right).

However, the OAM sorting has been limited only in the detection of the topological charge of the OVs. To overcome the limitation, a single azimuthal-quadratic metasurface for simultaneous SAM and OAM sorting has been proposed [88]. The metasurface is based on the photonic momentum transformation (PMT) principle [77], which allows OVs with different topological charges to focus on the same image plane with different azimuthal coordinates. Through this method, spin controlled multifunctional PMTs have been demonstrated for instantaneous SAM and OV sorting. This is accomplished by the dielectric metasurface that combines both the PB phase and propagation phase at the wavelength of 532 nm. As depicted in Figure 4h, LCP (*σ* = 1) and RCP (*σ* = −1) incident beam takes the spin-decoupled azimuthal-quadric phase profiles [77]. Thus, OV of various spin is mapped into the upper and lower halves of the observation screen. Consequently, SAM can be easily sorted by examining which half of the screen the focal spot is formed. For example, *σ* = 1 OVs can be seen in the upper half of the observation plane, while *σ* = −1 OVs appear in the lower half. Moreover, the topological charge can be recognized by measuring the azimuthal coordinate of the focal spot shown in Figure 4h.

### 4.3 OV multiplexing

**Figure 5:**
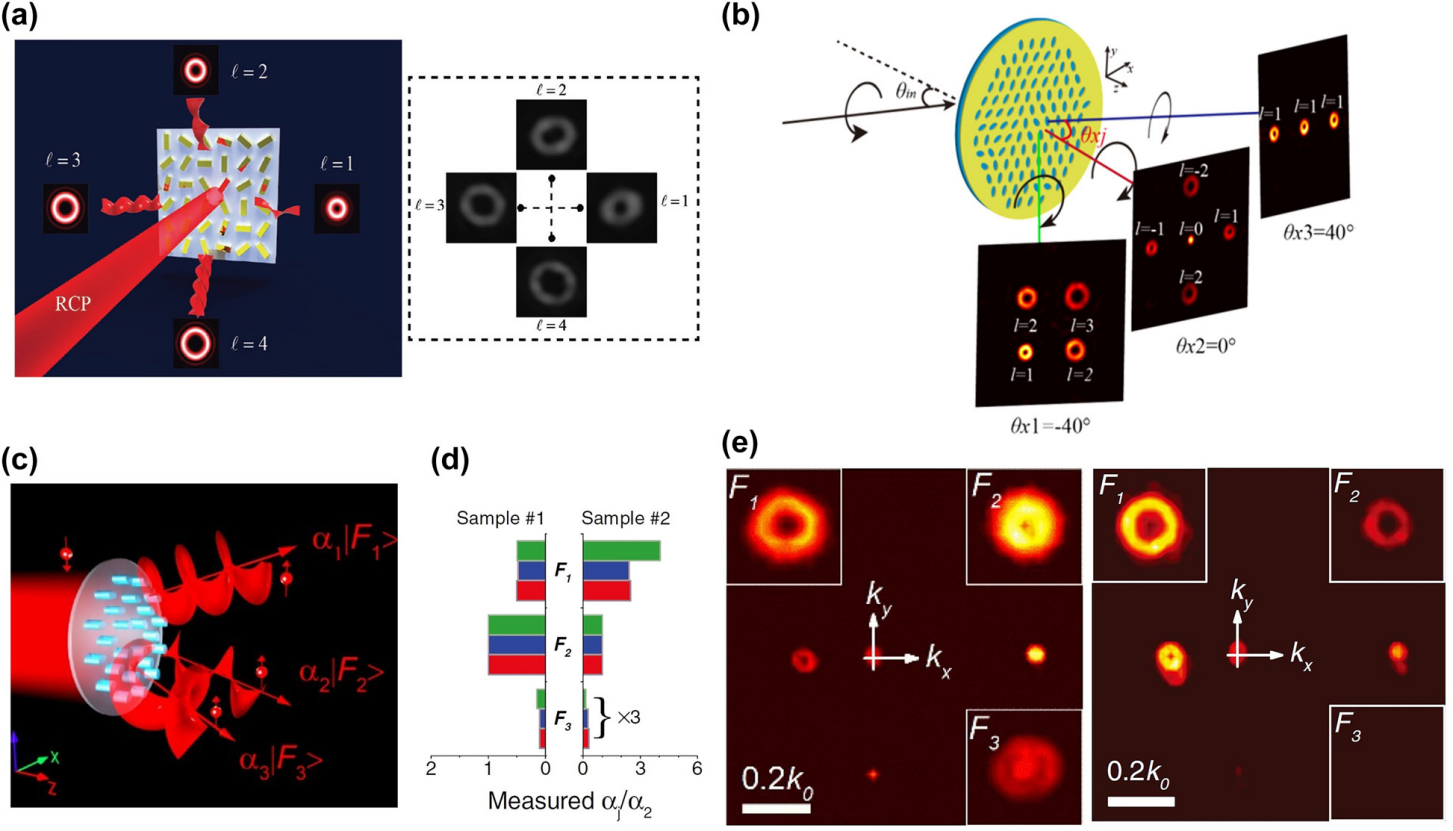
Metasurface for OV multiplexing. (a) Left: Schematic of off-axis multi-OAM generation. Right: Measured intensity profiles of four OAM beams with topological charges ranging from *l* = 1 to 4 generated under the illumination of an RCP light [80]. (b) Schematic of angle multiplexed multichannel OV generation based on the PB phased based metasurface. The light with different colours depicts different deflection angles. *θ*_*in*_ is the angle of incidence, while *θ*_*xi*_ (*i* = 1, 2, and 3) is the deflection angle of an outgoing light [81]. (c) Schematic of the energy tailorable multifunctional metasurface [82]. (d) The designed, calculated, and measured energies of each functionality of designed samples [82]. (e) The measured light intensity distributions of fabricated samples in *k*-space [82].

Enhancing the information-carrying capacity of incident light has always been a prime objective in both academia and industry. Multi-channel metasurfaces with different OAM states can facilitate high-capacity optical communication [79]. Recently, an enthralling approach to generate multiple OAM states and arbitrarily control their superpositions has been experimentally demonstrated [80]. A minimalist metallic metasurface (composed of gold nanorods) is used to realize OVs in four different channels with different topological charges. OV superposition is controlled in multi-channels by tunning the polarization of input light. This methodology can continuously manipulate various OV superpositions in multiple channels. To illustrate the versatility and high performance of this platform, a metasurface that can realize different hybrid superpositions of OAM states in four separate channels has been proposed (Figure 5a). Four OAM beams with different topological charges, ranging from *l* = 1 to 4 in separate channels, can be observed for an incident RCP Gaussian beam (Figure 5a). An OAM beam with a topological charge of *l* has a ‘doughnut’ intensity profile with a dark area in the beam center. The radius of ‘doughnut’ defined by the distance from the center to the maximum intensity points, is expressed by $r=\omega \sqrt{\left(l/2\right)}\text{,}$ where *ω* is the beam radius [80].

Besides, multi-channel OV arrays generators based on angular multiplexing have been proposed. Angular multiplexing of multi-channel OV arrays has been performed using elliptical nanohole-based single geometric metasurfaces [81]. The angular-multiplexed multi-channel OV arrays imply a sequence of OV arrays with various topological charges deflected in the azimuthal direction, as shown in Figure 5b. When a CP light at a certain angle impinges on the metasurface, different OV arrays deflect at various angles on the transmission side. The intensity distribution and topological charge of the OV array in each channel can be individually determined. The deflection angle of the OV array is closely related to impinging light angle, providing an extra degree of freedom by adding a new OV array by adjusting the illumination angle. This method can significantly enhance the information security and storage capacity of ultrathin optical devices. Ideally, the deflection angle and number of the OV array are unconstrained. Nevertheless, the size of the OV array, the limited angle range, the number of channels, and the crosstalk are the main deciding factors in the actual design process. Figure 5b shows three forms (2 × 2 OV array, rhombus OV array, and 1 × 3 OV array) of OVs arrays at three different channels. Moreover, to show the affability of the design, the topological charges in the second channel are spatially variant.

It is vital to precisely tailor the energy configuration of each channel in multi-channel OAM applications. Recently, Liu et al. proposed a design strategy to realize energy tailorable multifunctional metasurfaces based on the full Fourier components manipulation of the optical field [82]. By varying the size and the orientation angles of the amorphous silicon elliptic nanopillars, the full Fourier components (intensity and phase) of the diffraction field can be manipulated. Based on these nanopillars, metasurfaces that can convert incident CP light to its cross-polarized light of different functionalities with predefined energy ratios can be realized. As an example, spin-selective metasurfaces that can generate different OVs with different topological charges and controllable energy ratios have been experimentally realized (Figure 5c). The measured energy distributions of different OVs fit well with the designed values, as shown in Figure 5d and e. This method avoids intrinsic noises and complex optimization processes in realizing multi-channel OAM metasurfaces and provides a new solution for realizing energy controllable multifunctional OVs applications.

### 4.4 OV holography

**Figure 6:**
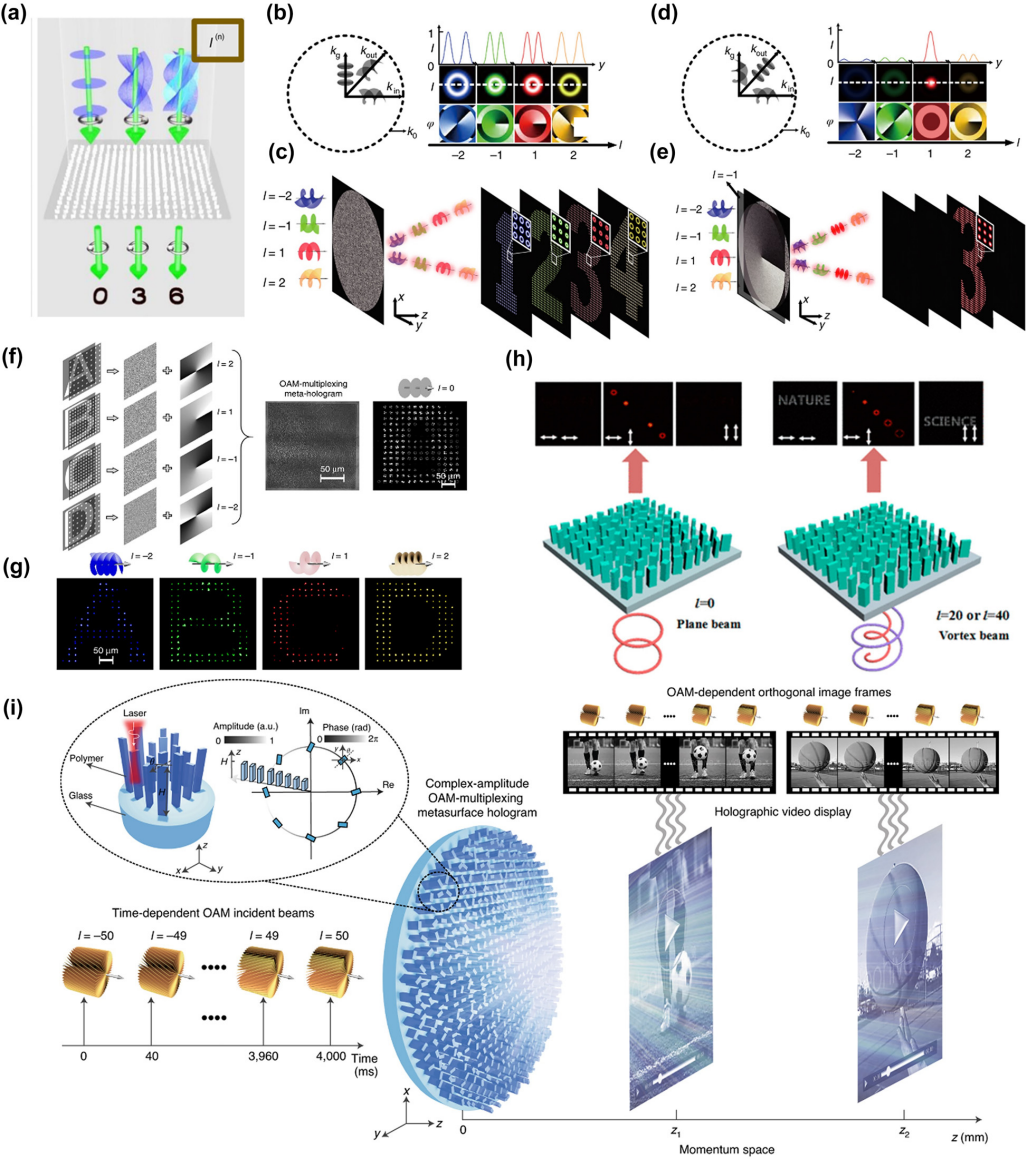
Metasurface for OV holography. (a) Meta-transformer utilizing OAM as an additional degree of freedom [97]. (b) Schematic of OAM property transfer in the spatial frequency domain (*k*-space) [97]. (c) OAM conserving meta-hologram that preserves the OAM property to the image plane [97]. (d) Schematic of OAM conversion after passing OAM selective hologram in the spatial frequency domain [100]. (e) OAM selective meta-hologram that constructs images at target OAM modes [97]. (f) Design of an OAM-multiplexing meta-hologram [99]. (g) Experimental result of an OAM-multiplexing meta-hologram [99]. (h) Vortex and polarization multiplexed meta-hologram [103]. (i) Schematic of complex-amplitude metasurface-based OAM-multiplexing hologram [101].

The metasurface holography is a powerful tool for optical encryption [83], [84], [85], [86], [87], [88], three dimensional (3D) displays [89], [90], [91], and data storage [91]. Metasurface holograms have been achieved by reconstructing the complex wavefronts of light with the technique of computer-generated holograms (CGH). For a higher capacity of information storage, various multiplexing techniques using wavelength [32, 92, 93], incident angle [94], [95], [96], and polarization [23, 53] have been explored. However, the hologram bandwidth has remained too low for practical use; the fact that OAM has an additional degree of freedom in unlimited OAM modes has raised interest in increasing the information capacity using OAM in metasurface holography. Therefore, holograms utilizing OAM as an additional information carrier have been proposed [97, 98]. For example, an OAM meta transformer has been developed by the multi-OAM phase retrieval algorithm to reconstruct holographic images ‘0’, ‘3’, and ‘6’ depending on the incident OAM modes *l* = 0, 3, 6, respectively (Figure 6a) [97]. However, conventional digital holography methods cannot fully utilize the diversity of OAM modes due to the lack of OAM mode selectivity.

Recently, a new concept of OAM metasurface holography utilizing the full capacity of OAM mode with a single hologram has been proposed [99], [100], [101]. The corresponding OAM metasurface hologram contains three types of hologram, including OAM conserving hologram, OAM selective hologram, and OAM multiplexing hologram [102, 103]. This concept paved the way for ultrahigh-capacity holographic multiplexing.

OAM conserved hologram is achieved by preserving the OAM property in each of the reconstructed hologram image pixels. To preserve the OAM property, the period of the discrete sampling array has been determined by the spatial frequency distribution of the incident OAM beam. The incident OAM beam with spatial frequency (*k*_*in*_) is linearly shifted in the OAM conserving hologram that is sampled by a spatial frequency (*k*_*g*_). Thus, the spatial frequency (*k*_*out*_) propagating outward from the metasurface has the same helical wavefront compared with the incident OAM beam (Figure 6b) [98]. A larger topological charge (*l*) of the beam results in a larger array period to prevent the spatial overlap of the helical wavefront kernel. As a result, the OAM property of the incident OAM beam is preserved in the reconstructed hologram as shown in Figure 6c [97].

By adding a spiral phase plate with a phase distribution of *lφ* (*k*_*g*_) to the metasurface for OAM conserving hologram, OAM selective hologram is achieved. In this metasurface, only an OAM beam with a topological charge of −*l* converts to a Gaussian mode due to the angular momentum conservation law (Figure 6d) [98]. Thus, number ‘3’ holographic image only appears when the OAM beam with topological charge of *l* = 1 is incident to the metasurface (Figure 6e) [99]. The reconstructed hologram pixels feature a solid-spot intensity distribution corresponding to the Gaussian mode.

OAM multiplexing hologram has been achieved based on the OAM selectivity. Four images are sampled in the spatial frequency domain, and each corresponding metasurface for OAM selective hologram is encoded with spiral phase plates with topological charges of *l* = 2, 1, −1, −2, respectively. Superposing these four phases for OAM selective holograms leads to the OAM multiplexing hologram (Figure 6f). Consequently, when the plane wave is incident to the metasurface, a complex interference pattern is constructed in the image plane, combining four alphabetic images for four distinct helical modes. However, when the OAM beam with varying topological charge is incident to the OAM multiplexing hologram, OAM-dependent distinct holograms are reconstructed in the image plane (Figure 6g) [99].

Furthermore, OAM and polarization multiplexed hologram has been demonstrated using an all-dielectric birefringent metasurface [99]. Utilizing the combination of the OAM states and the input, output polarization, different holograms are reconstructed in the image plane. When the plane wave Gaussian beam (*l* = 0) is illuminated to the metasurface, holographic images are hidden in the *T*_*xx*_ and *T*_*yy*_ channel (where the subscript designates the input and output polarization channels) and a spatially differing vortex beam profiles with topological charge only appears in the *T*_*xy*_ and *T*_*yx*_ channel (Figure 6h) [101]. The holographic image ‘nature’ and ‘science’ appears in the *T*_*xx*_ and *T*_*yy*_ channels when the illuminated beam with target angular momentum (*l* = 40 with *x-*polarization and *l* = 20 with *y-*polarization) is incident to the metasurface. This method further increases the information capacity of the OAM multiplexing holograms.

However, the OAM multiplexed holograms mentioned above has been achieved by using phase-only metasurfaces. Such phase-only metasurface hologram possesses the upper bound of the maximum number of OAM dependent holograms. Two critical reasons are the CGH retrieval algorithms of the conventional holograms and the spatial resolution limits in the fabrication of metasurface. The conventional CGH retrieval algorithms convert the complex-amplitude to the phase-only value, resulting in total loss of the amplitude information of each image channel; this creates substantial crosstalk in multiplexed images limiting the maximum number of holograms that can be multiplexed. A complex-amplitude metasurface has been proposed to fundamentally solve these limits of conventional phase-only OAM multiplexing holograms [101]. The complex-amplitude metasurface uses polymer-based meta-atoms that vary in height and rotation to cover the full range of the complex-amplitude information. The 3D laser printing technology based on two-photon polymerization induced by a femtosecond laser enables the production of height varying meta-atoms. Thus, the complex-amplitude metasurface successfully reconstructs two sets of hundred orthogonal OAM multiplexed images at two different planes in the momentum space (Figure 6i) [101], showing the potential of the OAM multiplexing meta-hologram as a high-capacity information storage and a future 3D holographic video display.

## 5 Nonlinear metasurfaces for OV generation and manipulation

**Figure 7:**
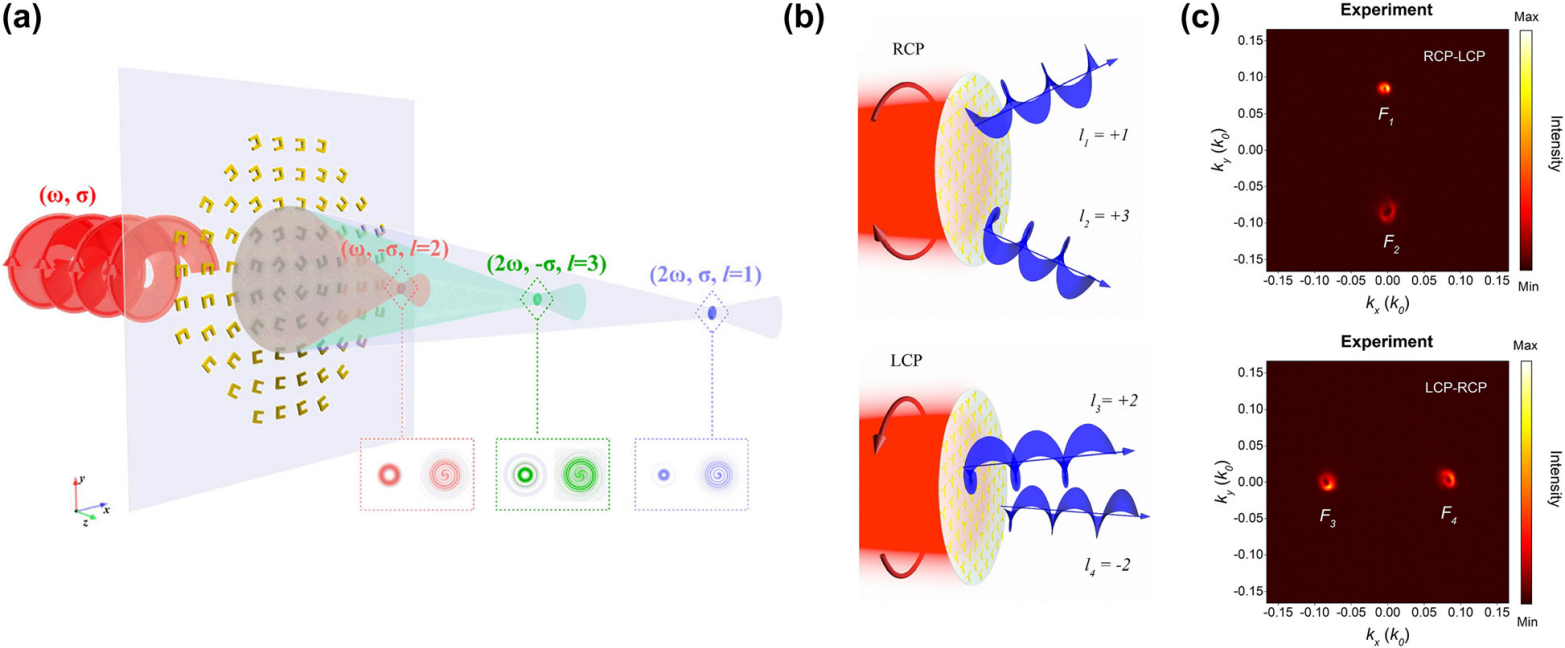
Nonlinear metasurface for OV generation and manipulation. (a) Schematic of the nonlinear multifunctional metasurface that can generate three focused OVs with different topological charges and different focal lengths [109]. (b) Schematic of the spin-controlled nonlinear metasurface that can generate nonlinear OVs with independent topological charges for LCP and RCP light [27]. (c) The measured LCP and RCP light intensity distributions of SHG signals in *k*-space for RCP and LCP incident light [27].

Recently, the nonlinear optical responses of metasurfaces have attracted more attention [104], [105], [106]. Nonlinear metasurfaces can provide more information channels and less background noise, thus, suitable for generating and manipulating OVs. Due to the nature of the PB phase [107], when an incident fundamental beam with CP state *σ* incidents on split ring resonators (SRRs) with an orientation angle of *θ*, the transmitted fundamental beam with opposite CP state will acquire the PB phase of 2*σθ*, and the second harmonic generation (SHG) signals with same or opposite CP states will acquire phases of *σθ* and 3*σθ*, respectively [108]. Therefore, three different phase profiles can be implemented in one metasurface simultaneously. Based on this method, Li et al. proposed a metasurface that can generate three focused OVs with different topological charges and different focal lengths [109]. As shown in Figure 7a, when the rotation angles of the SRRs change from 0 to 2*π* along the azimuthal direction, according to the principle of the PB phase, three OVs with topological charges of *l* = 1, 3, and 2 can be generated for the transmitted SHG beams with two different CP states and fundamental beam with converted CP state, respectively. At the same time, the three OVs can be focused on different focal planes by endowing different parabolic phase distributions along the radial direction of the metasurface. With the help of the nonlinear PB phase, this metasurface can triple the capacity of OVs, which can greatly increase the number of information channels. Based on a similar strategy, three nondiffracting Bessel beams with different topological charges can also be generated with one nonlinear metasurface [110]. It should be noticed that, due to the selection rules of harmonic generation processes, some harmonic signals cannot be generated by nanostructures with specific rotational symmetry [111, 112]. For example, nanostructures with three-fold rotational (C3) symmetry cannot generate second harmonic OVs with CP state same as that of the incident light [34]. In addition, these previous nonlinear metasurfaces cannot simultaneously manipulate the amplitude and phase of the harmonic wave signals, which hinders the development of nonlinear metasurfaces to more complex applications.

Recently, Hao et al. proposed a design strategy of nonlinear metasurfaces to realize the complex-amplitude modulation of the SHG [27]. Based on the hydrodynamic model of the free electron dynamics, they developed a method to quantitatively predict the amplitude and phase of the SHG signals radiated from the nanoparticle. Then, by varying the orientation angles and the size of the gold nanostructures with C3 symmetry, the complex-amplitude modulation of the SHG signals was realized. In addition, because they combined the nonlinear PB phase and the nonlinear resonance phase, the phase of LCP light and RCP light can be decoupled. Finally, a nonlinear spin-selective OAM multiplexing metasurface and a spin-controlled multifunctional metasurface that can generate nonlinear OVs with independent topological charges for LCP and RCP light were designed and experimentally realized, as shown in Figure 7b and c. By arbitrarily manipulating the amplitude and phase of nonlinear signals for arbitrary pairs of orthogonally polarized light, more complex OAM applications are foreseeable in the future.

## 6 Outlook and conclusions

A vortex beam carries the OAM, which has played an important role in fundamental research and practical applications due to the additional degree of freedom. The peculiar features of OAM beams have offered new opportunities for applications in optical communications, particle manipulation, and quantum science. Conventional optical systems consist of cascaded bulky optical elements, leading to large volume and high cost. In addition, the misalignment between light beams and OV beams generators results in poor purity of OAM modes (e.g., superposition of multiple OV beams). Despite the fact that conventional optical systems can generate and manipulate OVs, miniaturized OAM devices and on-chip devices are desirable to meet the continuing trend of system integration (e.g., optical fibers).

To keep pace with the continued miniaturization of devices and the daunting increase in the volume of information, novel approaches to process and transmit information are required. To address these requirements, optical metasurfaces have provided a new paradigm in OAM based on-chip photonic devices by engineering and exploiting the unprecedented optical properties of novel metasurfaces. In addition to their unique and diverse optical properties, optical metasurfaces possess unprecedented material compatibility and ease of integration with integrated photonic chips. Low-cost integrated photonic chips can easily and efficiently combine functionalities, as demonstrated in the last few years by emerging low-loss silicon photonic chips, with which highly integrated optical interconnects operating at terabit-per-second speeds are within reach. The emergence of optical metasurfaces represents a paradigm shift to achieve an efficient approach incorporated into complex photonic circuits on-chip to allow market-scalable quantum technologies. This will potentially lead to new generation OAM sources that are very difficult or challenging to realize with conventional optics and allow for multiplexing numerous sources on a small footprint.

For future OV-related research, fundamental advances in optical metasurfaces and light-matter interactions have a major impact. For example, the recent work in complex-amplitude OAM multiplexing meta-hologram that use height varying meta-atoms fabricated by 3D laser printing technology allow metasurface to preserve the amplitude information of each image pixel and drastically increase the number of OAM multiplexing images [99]. By decreasing the crosstalk and further increasing the number of OAM multiplexing images, OAM multiplexing meta-hologram can serve as large-capacity information storage, optical encryption, and future realistic 3D display. The energy tailorable multi-channel OAM metasurfaces and multifunctional nonlinear OAM metasurfaces demonstrate the potentials of metasurfaces in manipulating the amplitude, phase, and frequency of OVs [113, 114]. The multidimensional manipulation of OVs based on metasurfaces is a key research direction in OVs research, which will bring numerous novel functionalities and applications in the future. Besides, dynamically reconfigurable OAM metasurfaces are also highly desirable for the realization of advanced OVs devices in practice. One promising way to reach this goal is to use active functional materials. For example, dynamically tunable ultrahigh order OVs have been proposed based on graphene metasurfaces [115], which can be easily controlled by varying the Fermi energy of graphene via electrostatic gating. However, there is a long way to go for the realization of real-time control of OVs and their practical applications.
